# Investigation of Electroacupuncture and Manual Acupuncture on Carnitine and Glutathione in Muscle

**DOI:** 10.1093/ecam/nep071

**Published:** 2011-06-15

**Authors:** Shizuo Toda

**Affiliations:** Department of Health Sciences, Kansai University of Health Sciences, 2-11-1 Wakaba, Kumatori, Sen-nan, Osaka-fu 590-0482, Japan

## Abstract

Electroacupuncture (EA) and manual acupuncture (MA) have therapeutic effects on muscle fatigue in muscle disease. The deficiencies of carnitine and glutathione induce muscle fatigue. This report investigated the effects of EA and MA on carnitine and glutathione in muscle. After the mice of EA group were fixed in the animal cage, right Zusanli (ST36) and Jiexi (ST41) were acupunctured and stimulated with uniform reinforcing and reducing method by twirling the acupuncture needle for 15 min. And then, the needle handles were connected to an electric stimulator for stimulating the acupoint with dense-sparse waves. After the mice of MA group were fixed in an animal cage, right ST36 and ST41 were acupunctured and allowed for 15 min. The mice of normal control group were not acupunctured and stimulated for 15 min. The mice of all groups were killed for collecting muscle tissue 1 h after the final treatment. Carnitine and glutathione in homogenate of muscle tissue were determined with carnitine (Kainos Laboratories Co., Tokyo, Japan) and glutathione assay kit (Dojin Chemicals Co., Kumamoto, Japan). Carnitine level in muscle tissue of MA group was significantly higher than those of EA group and normal control group. Carnitine level in muscle tissue of EA group was not significantly different from that of normal control group. Glutathione levels in muscle tissue of EA group and MA group were significantly higher than that of normal control group. This report presented that carnitine in muscle is increased by MA, and not increased by EA, and that glutathione in muscle is increased by EA and MA.

## 1. Introduction

Muscle disease are associated with contractures, craps muscle stiffness and deep muscle aching. These phenomena relate to muscle fatigue [1].

The pathogenesis of fatigue in muscle condition has the relation carnitine and glutathione. Carnitine appears to improve force of muscle while stimulated *in situ*. This effect of carnitine is acute and stereospecific [2]. In energy metabolism, carnitine has a major role in the translocation of long-chain fatty acids into the mitochondrial matrix for subsequent *β*-oxidation, and in the regulation of the mitochondrial in skeletal muscle [3]. The alternative explanation on skeletal muscle fatigue has been considered the effects of reactive oxygen species (ROS). ROS scavenger as glutathione reduces skeletal muscle fatigue [4].

Acupunctures as electroacupuncture (EA) and manual acupuncture (MA) can protect cells from injury of acute spots and maintain the functions of mitochondria so as to delay fatigue, prolong working time of muscles [5]. It has been unclear the difference of effect between EA and MA on muscle.

This report investigates the effects of EA and MA on carnitine and glutathione in muscle.

## 2. Materials and Methods

### 2.1. Grouping of Animals

Male ddY mice weighing between 25 and 30 g, 6- to 7-weeks-old were obtained from SLC, Shizuoka, Japan. They were housed in a room maintained at 25°C with a relative humidity of 60% and given standard laboratory chow and water *ad libitum* in the Laboratory Animal Center of Kansai University of Health Sciences. The care and use of animals followed the guidelines of Laboratory Animals in Kansai University of Health Sciences. Fifteen rats were allocated to three groups. They were EA, MA and normal control groups. There were five mice in one group.

### 2.2. Acupoint and Method of Acupuncture

The animal cage (Orientalgiken Co., Tokyo, Japan) has five holes for four limbs and a tail. During acupuncture and stimulation, mice were maintained in the animal cage with right hind limbs taken out and fastened to the wall of the animal cage with tape. In EA group and MA group, the mice were acupunctured by inserting 3 mm deep at right Zsusanli (ST36) and Jiexi (ST41) with sterilized disposable stainless steel acupuncture needles (0.25 × 30 mm, Seirin Co., Shizuoka, Japan). Mice ST36 were determined according to human ST36 which is located on the tibialis anterior muscle [6]. Mice ST41 were determined according to human ST41 which is located between two tendons on the dorsum of the foot which are more distinct when the ankle is dorsiflexion, and is the midpoint of the line connecting the prominences of the lateral malleolus and medical malleolus [6].

After the mice of EA group were fixed in the animal cage, right ST36 and ST41 were acupunctured and stimulated with uniform reinforcing and reducing method by twirling the acupuncture needle for 15 min. And then the needle handles were connected to Neurostimulator (Neuro Medical Co. Osaka, Japan) for stimulating the acupoints with dense-sparse waves, frequency of 3 Hz for 30 min, and amplitude: positive pulse 50 V; negative pulse 25 V.

After the mice of MA group were fixed in the animal cage, right ST36 and ST41 were acupunctured and allowed for 15 min.

The mice of normal control group were not both acupunctured and stimulated.

### 2.3. Collection of Muscle Samples and Measurements of Carnitine and Glutathione

The mice of EA group and MA group were killed for the treated skeletal muscles between lateral ST36 and ST41 1 h after the treatment. The treated skeletal muscles were removed from the mice immediately after killing and were subjected to homogenization in ice-cold 50 mM Tris–HCl buffer at pH 7.4 using a Potter-Elvehjem homogenizer. Carnitine and glutathione in homogenate of muscle tissue were determined with carnitine (Kainos Laboratories Co., Tokyo, Japan) and glutathione assay kit (Dojin Chemicals Co., Kumamoto, Japan).

### 2.4. Statistical Analysis

Data were expressed as mean ± standard error and analyzed by Mann-Whitney *U*-test.

## 3. Results

Carnitine level in muscle tissue of MA group was significantly higher than those of EA group and normal control group (*P* < .001). Carnitine level in muscle tissue of EA group was not significantly different from that of normal control group. Glutathione levels in muscle tissues of EA group and MA group were significantly higher than that of normal control group (*P* < .001) (Table 1).

## 4. Discussion

Acupuncture at ST36 and ST41 has been shown to be a better therapeutic effect for muscle fatigue, problems of constriction and myofacial pain of leg muscle [7]. Acupuncture at these acupoints reduces the tibialis anterior electromyographymuscle activity [8]. Acupuncture leads to analgesia and improvement in other somatic sympotoms of fibromyalgia on a single-site, single-blind, randomized trial [9]. Acupuncture has been useful therapy on muscle symptoms.

Carnitine therapy is effective in ameliorating fatigue in celiac disease on randomized double-blind versus placebo parallel study [10]. Carnitine can directly improve the fatigue characteristics of muscles enriched in type I fiber [11]. More than 95% of carnitine in the body exists within skeletal muscle tissue [12]. Carnitine transfer fatty acids to the mitochondria and involves energy metabolism. Carnitine deficiencies induce muscle fatigue and myopathy [13]. Carnitine in blood and brain showed to be increased by acupuncture [14]. Contraction-induced production of ROS causes oxidative stress to skeletal muscle [15]. ROS contribute to muscle fatigue. Anti-oxidant as glutathione has been shown to lessen oxidation of cellular constituents and delay muscle fatigue [16]. The addition of anti-oxidant as GSH can improve muscle performance [17]. EA has the protective effects on mitochondria of cortical neuron in acute cerebral ischemia-reperfusion rats. It has been speculated that these results are related with anti-oxidant as glutathione [18]. These following findings showed that the effects of acupuncture may be related with carnitine and glutathione.

EA and MA can increase Ca^2+^ content and Ca^2+^-ATPase activity in sarcoplasmic reticulum of skeletal muscle cells in motor fatigue rats [19]. EA is superior to MA in training patients with tennis elbow [20]. These findings showed that these differences of effects between EA and MA may depend on the condition of muscle.

The presented results suggested that increases of carnitine by MA and of glutathione by EA and MA may improve muscle disease. The differences of carnitine level between EA and MA may depend on acupuncture stimulation. This investigation should be progressed further.

## 5. Conclusion

EA and MA have been shown to have therapeutic effects on muscle fatigue in muscle disease. This report presented that carnitine in muscle is increased by acupuncture, and not increased by EA, and that glutathione in muscle is increased by EA and MA.

## Figures and Tables

**Table 1:** Comparison of carnitine and glutathione contents in muscle tissues between three groups (mean ± standard error, *μ*mol/g).

Groups	Carnitine	Glutathione
Electrocupuncture group	363.33 ± 46.33	34.11 ± 2.03^a^
Manual acupuncture group	654.17 ± 50.52^b,a^	31.38 ± 1.34^a^
Normal control group	346.67 ± 53.41	26.79 ± 3.18

^a^ *P* < .001 versus normal control group; ^b^ *P* < .001 versus electroacupuncture group.
